# The Importance of TOR Kinase in Plant Development

**DOI:** 10.3389/fpls.2020.00016

**Published:** 2020-02-05

**Authors:** Kirsty McCready, Victoria Spencer, Minsung Kim

**Affiliations:** School of Biological Sciences, Faculty of Biology, Medicine and Health, University of Manchester, Manchester, United Kingdom

**Keywords:** TARGET OF RAPAMYCIN (TOR), plant development, nutrient sensing, meristem patterning, leaf development, flower induction

## Abstract

TARGET OF RAPAMYCIN (TOR) kinase has been recognised as a key developmental regulator in both plants and animals. Despite their distinct developmental programmes, all eukaryotes studied possess a functional TOR kinase, which integrates environmental and nutrient signals to direct growth and development. This is particularly important in plants, as they are sessile and must sense and respond to external signals to coordinate multicellular growth appropriately. Thus, the investigation of TOR is essential for plant developmental studies in the context of the resources available for growth. Recently, links have been shown between TOR and plant development from embryogenesis through to senescence, however more investigation is crucial to fully elucidate TOR function in each developmental process.

## TOR Is a Key Plant Developmental Regulator

Mounting evidence suggests that integrated signaling and metabolic networks play an instructive role in developmental programs and responses to environmental changes and stresses (Li and Sheen, 2016; Krejci and Tennessen, 2017). Remarkably, the TARGET OF RAPAMYCIN (TOR) protein kinase has been identified as a “master regulator” of such networks in all eukaryotes, from single-celled yeast and algae, to complex multicellular organisms such as plants, animals and humans (Dobrenel et al., 2016). Nutrients and growth factors activate TOR, whilst energy deprivation, starvation and stresses are responsible for its inactivation (Dobrenel et al., 2016).

Since the discovery of the TOR inhibitor rapamycin from the soil bacterium *Streptomyces hygroscopicus* (Sehgal et al., 1975), and its use to identify and isolate *TOR* in yeast (Heitman et al., 1991; Kunz et al., 1993), mammals (Sabatini et al., 1994) and plants (Menand et al., 2002), our knowledge and understanding of TOR signaling mechanisms and function has progressed immensely. Nevertheless, the study of plant TOR has been largely limited to the model plant *Arabidopsis thaliana* (and select few other plant species, see: Nanjareddy et al., 2016; De Vleesschauwer et al., 2018) and further investigation is crucial if we are to fully elucidate TOR function in diverse developmental processes across the plant kingdom.

## The Plant TOR Kinase Complex

A single large *TOR* gene exists in Arabidopsis, *Chlamydomonas reinhardtii*, most animals and humans (Xiong and Sheen, 2014). *TOR* encodes a highly conserved Ser/Thr kinase (Menand et al., 2002; Zoncu et al., 2011), belonging to the phosphatidylinositol 3-kinase-related kinase family (Heitman et al., 1991). In plants, TOR functions as a complex [TARGET OF RAPAMYCYIN COMPLEX1 (TORC1)] with the core components REGULATORY-ASSOCIATED PROTEIN OF TOR (RAPTOR) and LETHAL WITH SEC THIRTEEN 8 (LST8) (Dobrenel et al., 2016). Whether other plant specific components exist remains to be determined. All sequenced plant species possess orthologs of the *RAPTOR* and *LST8* genes (Anderson et al., 2005; Deprost et al., 2005; Duan et al., 2006; Mahfouz et al., 2006; Diaz-Troya et al., 2008; Moreau et al., 2012).

Two homologs of the mammalian *RAPTOR* gene exist in Arabidopsis, *RAPTOR1* (or *RAPTOR1B*, AT3G08850) and *RAPTOR2* (or *RAPTOR1A*, AT5G01770) (Anderson et al., 2005; Deprost et al., 2005). *In silico* analyses reveal that *RAPTOR1* is highly expressed throughout development, whereas *RAPTOR2* expression is markedly lower. As there is only one *RAPTOR* gene in algae (Diaz-Troya et al., 2008), it has been suggested that *RAPTOR2* arose by a duplication of the ancestral *RAPTOR* gene in the land plant lineage and is a redundant copy (Deprost et al., 2005), however more detailed *RAPTOR* phylogenies are needed to test when this occurred. In some reports *raptor2* mutants display no obvious phenotypic defects (Deprost et al., 2005), further supporting redundancy, however a slight increase in autophagy was detected in Arabidopsis seedlings and protoplasts (Pu et al., 2017). The protein structure of RAPTOR is conserved in plants; RAPTOR1 has three HEAT motifs followed by seven WD-40 repeats, which are important for protein interactions (Deprost et al., 2005). The conservation of the TOR and RAPTOR1 interaction *via* the TOR HEAT motifs has been confirmed by coimmunoprecipitation experiments in tobacco leaves (Mahfouz et al., 2006), however higher resolution imaging of the complex would be useful to compare to recent electron microscopy studies of TORC1 and TORC2 in mammals and yeast to study structural conservation (Adami et al., 2007; Aylett et al., 2016; Karuppasamy et al., 2017). Future work into plant-specific RAPTOR interactions in different tissues would also prove informative for elucidating any direct interactions with plant development pathways.

All of the plant genomes checked contain an *LST8* gene, however two *LST8* genes (*LST8-1* and *LST8-2*) have been found in *Arabidopsis thaliana* and *A. lyrata* as a result of a gene duplication event in their common ancestor (Moreau et al., 2012). As in other eukaryotes, the Arabidopsis LST8-1 (AtLST8-1) protein contains seven WD-40 repeats. GUS reporter expression analyses reveal that *LST8-1* is expressed throughout plant development, particularly in the aerial tissues. Yeast and Arabidopsis LST8 proteins share 51% sequence identity, and yeast expressing the *AtLST8-1* coding sequence were able to grow normally, demonstrating that *AtLST8-1* is a homolog of yeast *LST8* with conserved function. As with RAPTOR, the interaction of LST8 with plant-specific components in different tissues will reveal potential pathways by which developmental phenotypes arise.

## TOR Function During Plant Development

Phylogenetic studies show that *TOR*, *RAPTOR* and *LST8* gene trees are congruent with the land plant species tree (Deprost et al., 2005; Moreau et al., 2012; Sapre et al., 2018), suggesting that this protein complex is highly conserved and therefore likely to be very important across the plant kingdom. However, how TORC was recruited during the evolution of multicellularity and plant specific processes is unclear. As well as controlling photosynthesis, autophagy and senescence (Deprost et al., 2007; Liu and Bassham, 2010; Moreau et al., 2012; Ren et al., 2012; Xiong et al., 2013; Li et al., 2015), TOR is critical for promoting different aspects of plant development under favourable conditions throughout a plant's lifespan.

### Embryogenesis

In flowering plants, seed formation is characterized by double fertilization of the female gametophyte, giving rise to two distinct tissues: the zygote and the endosperm (Dumas and Rogowsky, 2008). The endosperm grows as a syncytium until it reaches around 200 nuclei, before cellularization. The Arabidopsis loss of function KO *tor* mutant endosperm reaches approximately 48 (± 13) nuclei and cellularization does not occur (Menand et al., 2002). Embryos of Arabidopsis null *tor* mutants arrest early at the dermatogen stage, with cells in metaphase still present. While cell division itself is thus not inhibited by the disruption of *AtTOR* in the embryo, cell growth is supressed (Menand et al., 2002). This is consistent with wide-scale downregulation of translation machinery and cell wall modifying enzymes such as *CELLULASE SYNTHASE 6* (*CESA6*) and *EXPANSIN B1* (*EXPB1*) after *AtTOR* inhibition (Xiong et al., 2013). On the other hand, the role of *RAPTOR1* in embryogenesis is unclear. *raptor1* T-DNA insertion lines had viable embryos, suggesting that *AtTOR* function in embryogenesis is independent of *RAPTOR1* (Anderson et al., 2005). However, further work found the same line (SALK_078159) to be embryo lethal (Deprost et al., 2005), therefore varying light and temperature growth conditions could affect the phenotypic severity.

### Germination

AtTOR has been implicated as a key mediator of environmental signals with seed germination (Xiong et al., 2013). To drive the transition from heterotrophic to photoautotrophic growth in Arabidopsis seedlings, glucose‐AtTOR signaling activates broad gene sets involved in the cell cycle and anabolic processes, and suppresses gene sets controlling catabolic processes (Xiong et al., 2013). This in turn activates root growth *via* glycolysis-mitochondria-ETC (electron transport chain) relays (Xiong et al., 2013). Furthermore, photosynthesis-derived sugars are necessary for hormones (auxin, brassinosteroid, cytokinin, and gibberellin) to promote rapid root elongation and reactivate the quiescent root during this transition to photoautotrophy (Xiong et al., 2013).

Two independent *raptor1* mutants (SALK_101990 and SALK_022096) had seeds with delayed germination and reduced stress resistance, resulting in reduced viability (Salem et al., 2017). Furthermore, seed-coat pigmentation and mucilage production was reduced, accompanied by changes in metabolic content, such as increased free amino acids, and decreased protective secondary metabolites and storage proteins. This is consistent with the transcriptional reprogramming of gene sets involved in central and secondary metabolism in response to glucose-AtTOR signaling in seedlings (Xiong et al., 2013). There were also increases in abscisic acid, auxin and jasmonic acid, all known to inhibit germination.

### Seedling Development

The transition from dark-grown (etiolated) to light-grown (de-etiolated) seedlings is accompanied by several morphological changes; elongation rate is reduced, the apical hook opens, true leaves undergo expansion and mature chloroplasts develop. The inhibition of AtTOR in seedlings, *via* either asTORis (active site ATP-competitive TOR inhibitors) or genetic suppression, reduces cotyledon greening, chloroplast development and seedling growth (Deprost et al., 2007; Dong et al., 2015; Li et al., 2015; Xiong et al., 2017). 40S ribosomal protein S6 KINASE (S6K) is a phosphorylation target of TOR, and promotes chloroplast development and seedling growth *via* the regulation of BR INSENSITIVE 2 (BIN2) (See review: Shi et al., 2018).

**Figure 1:**
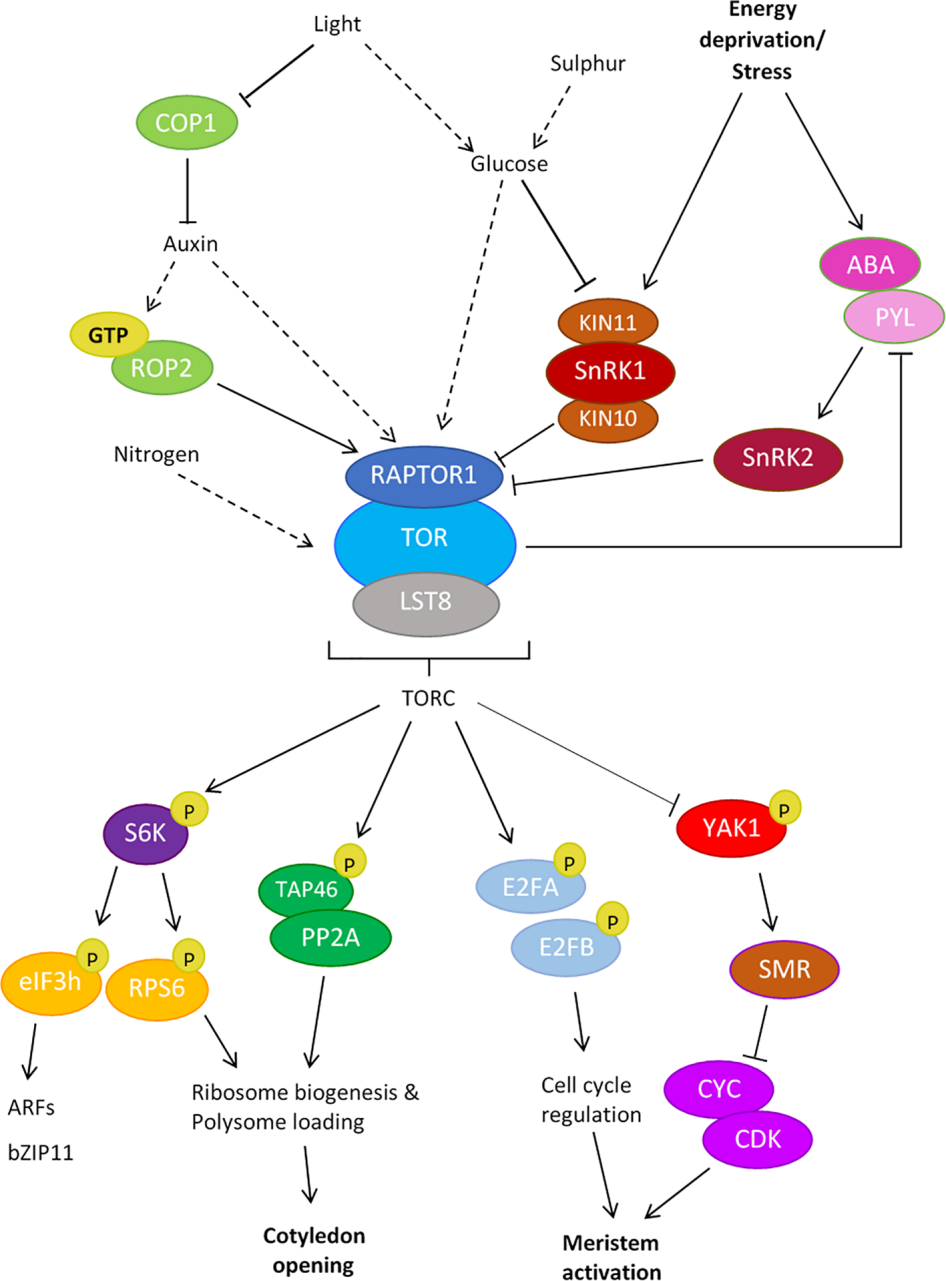
Upstream and downstream targets of the TOR Complex (TORC) in plants. *Upstream regulators of plant TORC1*: Light, glucose and nutrients are known activators of the TOR pathway. Light activates the TOR pathway *via* the inactivation of the negative regulator COP1, triggering the activation of the auxin pathway, leading to TOR activation during seedling de-etiolation. Light is also known to trigger GTP-ROP2/auxin activation of TOR signaling in the shoot apex. Light and glucose coordinate to inactivate the TOR antagonist SnRK1, leading to the indirect activation of TORC. The TORC kinase also senses sulfur availability indirectly through glucose signaling; sulfur deficiency induces low glucose levels, leading to the negative regulation of TOR signaling. Stress signals trigger ABA binding of PYL receptors, which activate SnRK2s. SnRK2s directly phosphorylate RAPTOR1, inhibiting TORC signaling to promote stress responses. *Downstream targets of plant TORC1*: Direct phosphorylation targets of TORC1 include PP2A (*via* the subunit TAP46), E2FA/B and S6K, leading to the activation of cellular processes throughout development. elF3H and RPS6 are direct phosphorylation targets of S6K-P. The YAK1 kinase is inhibited by active TOR, relieving the inhibition of CYC/CDKs by SMRs to allow cell proliferation in the meristem. Solid arrows indicate direct interaction, dashed arrows indicate indirect interaction.

An exciting link has been made between light and the activation of AtTOR-RPS6 (RIBOSOMAL PROTEIN S6) in de-etiolating seedlings. Light is first perceived by photoreceptors such as phytochrome A and cryptochromes, leading to the inactivation of the negative regulator CONSTITUTIVE PHOTOMORPHOGENESIS 1 (COP1), which triggers the activation of the auxin pathway and thus AtTOR-dependent phosphorylation of RPS6 (**Figure 1**). Accordingly, mutant seedlings lacking functional AtTOR, RPS6A or RPS6B displayed delayed cotyledon opening (Chen et al., 2018). It has also been shown that auxin can activate TOR *via* Rho-like small GTPase 2 (ROP2) (Schepetilnikov et al., 2013; Schepetilnikov et al., 2017). TOR may therefore coordinate light and auxin levels to ensure a timely switch in the development of de-etiolating seedlings.

### Meristem Development

The Shoot Apical Meristem (SAM) and the Root Apical Meristem (RAM) maintain undifferentiated stem cells responsible for the formation of the above- and below-ground organs, and *AtTOR* is known to be expressed in these tissues (Menand et al., 2002). Consistently, delayed shoot growth has been observed in *tor* knockdown and *raptor1* mutant lines (Menand et al., 2002; Xiong et al., 2013; Pfeiffer et al., 2016). Recent data showed that in 4-5 day old Arabidopsis seedlings, AtTOR-E2FA phosphorylation activates the RAM by activating S phase, whilst AtTOR, activated by light-Auxin-ROP2 signaling, phosphorylates both E2FA and E2FB to activate S phase in the SAM (**Figure 1**; Xiong et al., 2013; Li et al., 2017). AtTOR is thus a likely candidate for the integrator of environmental signals from distant organs to direct meristem activity in both roots and shoots.

YET ANOTHER KINASE 1 (YAK1) has recently been reported as a downstream target of the AtTOR pathway and a major regulator of RAM activity (Barrada et al., 2019). YAK1 was discovered through a pharmaco-genetic screen; *yak1* loss-of-function mutants are resistant to the asTORis AZD-8055, whilst Arabidopsis overexpressing *YAK1* are hypersensitive. Treatment of WT plants with AZD-8055 reduces the number of dividing cells in the meristematic zone (MZ) inducing early differentiation. In *yak1* mutants MZ size was not reduced in the presence of the inhibitor. Furthermore, when treated with pINDY, an ATP-competitive inhibitor of the animal AtYAK1 homolog DYRK1A (DUAL SPECIFICITY TYROSINE PHOSPHORYLATION REGULATED KINASE 1A), MZ size was restored in WT plants treated with AZD-8055. These results indicate that YAK1 controls cell proliferation in the MZ in a TOR-dependent manner.

Cell proliferation is controlled by CYCLIN DEPENDENT KINASES (CDKs), which are regulated by the periodic activation of cyclins (CYC). CDK-CYC activity is regulated by plant-specific CDK inhibitors such as SIAMESE (SIM) and SIAMESE-RELATED (SMR) (See review: Inagaki and Umeda, 2011). Various promoter-driven GUS reporter lines tracking the expression of *SIM*, *SMR*, and *CYC* genes revealed that, in the absence of AtTOR activity, YAK1 induces the expression of *SMR* genes, which in turn repress CDKs to promote differentiation (Barrada et al., 2019). Contrarily, YAK1 inhibition by AtTOR kinase promotes growth, by lifting the repression of CDKs and CYC, to maintain proliferation (**Figure 1**). Furthermore, physical interaction between AtYAK1 and RAPTOR1 has been confirmed by both yeast two-hybrid assay and biomolecular fluorescence complementation (BiFC) assays in plant cells (Forzani et al., 2019). A phosphoproteomics analysis in growth-induced Arabidopsis cell culture further demonstrated TOR-dependent phosphorylation of two conserved Ser residues of AtYAK1 (Van Leene et al., 2019). It has thus been suggested that TORC1 binds to AtYAK1 through the component RAPTOR and inactivates it by phosphorylation (Forzani et al., 2019), perhaps revealing how the TOR-YAK1-SMR-CYC/CDK interaction functions to regulate RAM activity and maintenance.

Patterning of the SAM by *WUSCHEL* (*WUS*) and *CLAVATA* (*CLV*) genes has also been connected to AtTOR activity (See review: Somssich et al., 2016). Interestingly, when three day old Arabidopsis seedlings were grown in AZD-8055, the activity of the *WUS* promoter in *pWUS::3xVENUS-NLS* lines decreased (Pfeiffer et al., 2016). *AtTOR* may therefore activate *WUS* expression, promoting meristem activity in favourable conditions. However, these seedlings were grown for three days in AZD-8055 liquid culture, so it is unclear to what extent long term metabolic changes are controlling *WUS* expression. Conversely, no expression changes of the root meristem patterning gene, *WUSCHEL RELATED HOMEOBOX 5* (*WOX5*) were observed when treated with asTORis, suggesting that *AtTOR* may not regulate meristem patterning in the RAM (Xiong et al., 2013). Further determining the exact role of AtTOR in the SAM and RAM will prove critical for understanding environment dependent meristem activity.

### Plant and Leaf Size

*AtTOR* is also involved in size regulation. ß-estradiol inducible and ethanol inducible *AtTOR* silencing plants show a reduction in plant biomass, including reduced cell size and ultimately reduced leaf size (Deprost et al., 2007; Xiong and Sheen, 2012), consistent with a T-DNA *raptor1* SALK line (Anderson et al., 2005). Accordingly, *lst8-1* mutants have reduced size, as well as increased shoot branching (Moreau et al., 2012). Ser-Thr PROTEIN PHOSPHATASE 2A (PP2A) contains a conserved regulatory subunit TAP46 (TYPE 2A-PHOSPHATASE-ASSOCIATED PROTEIN 46KD) (TAP42 in yeast), which is directly phosphorylated by AtTOR (**Figure 1**; Ahn et al., 2011). Disruption of *TAP46* expression results in global translation defects, decreased polysome accumulation and methionine incorporation, and in turn smaller plants as above (Ahn et al., 2015). Furthermore, a recent study has confirmed that *TOR* inhibition with asTORis prevents leaf primordia initiation in 10 day old Arabidopsis plants, causing a reduction in leaf number (Mohammed et al., 2018).

Conversely, overexpression of both *TAP46* and *AtTOR* results in larger seeds and plants (Deprost et al., 2007; Ahn et al., 2015), with bigger leaves due to larger epidermal cells and longer petioles. *AtTOR* domain overexpression lines possess twisted leaves and siliques (Deprost et al., 2007; Ahn et al., 2011). Together these studies clearly indicate the involvement of AtTOR in leaf development, however it is unclear whether *AtTOR* only directly controls global cell cycle regulators and cell growth machinery (Xiong et al., 2013; Li et al., 2017), or affects leaf development genes such as the *OVATE FAMILY PROTEINS* (*OFPs*) (Wang et al., 2011) to target specific leaf development pathways.

Nutrients such as nitrogen (N), phosphate (Pi), and sulfur (S) play crucial roles in the promotion of plant growth and recent studies suggest that AtTOR functions in these processes. For example, S availability coordinates glucose signaling to activate AtTOR (**Figure 1**; Dong et al., 2015). Furthermore, nitrate, a major N source, behaves as a nutrient signal to promote system-wide shoot and root growth in Arabidopsis (Liu et al., 2017). Notably, Arabidopsis seedlings modified to overexpress *AtTOR* show hypersensitivity to high nitrate inhibition of roots (Deprost et al., 2007). By sensing the nutrient content in the cell, AtTOR kinase is able to initiate growth at a time when sufficient resources are available for healthy plant development.

Abscisic acid (ABA) signaling has been implicated as a critical player in the inhibition of plant growth under stress and recent studies propose TOR is the key mediator of this process (Wang et al., 2018). Upon stress induction, ABA binds PYR1/PYL/RCAR (PYL) receptors, triggering the activation of SnRK2s (SUC NON-FERMENTING 1-RELATED KINASE 2). SnRK2s phosphorylate RAPTOR, thereby inhibiting TORC signaling and promoting stress-induced growth inhibition. When favourable conditions return, TOR phosphorylates PYL receptors, preventing ABA binding, and, critically, inhibiting the activity of ABA-independent PYLs. This interaction between ABA core signaling components and TORC represents a conserved regulatory mechanism to maximise fitness under stress and promote growth recovery in its absence.

Other regulators of plant growth *via* TOR include SnRK1, a conserved glucose/energy sensor protein kinase. Glucose can activate AtTOR indirectly *via* the inactivation of SnRK1 (**Figure 1**; Baena-Gonzalez and Sheen, 2008). Arabidopsis SnRK1 (AtSnRK1) forms a heterotrimeric complex with the catalytic subunits KIN10 and KIN11 (Baena-Gonzalez and Sheen, 2008), and KIN10 has been shown to directly interact with and phosphorylate RAPTOR (Nukarinen et al., 2016). Thus, TORC1 and AtSnRK1 dominate a complex network, acting antagonistically to direct plant growth.

### Flowering

Alongside altering organ size and initiation, *AtTOR* disruption delays flowering time (Deprost et al., 2007), which is also evident in *raptor1* and *lst8-1* mutants (Anderson et al., 2005; Moreau et al., 2012). The transition to flowering time is controlled by myriad external and internal factors, such as plant age, sugar availability, photoperiod and temperature (See review: Cho et al., 2017). These signals converge on factors such as *LEAFY* to convert the SAM into an Inflorescence Meristem (IFM) (Blazquez et al., 1997). Future work is necessary to determine whether AtTOR interacts with these pathways directly or indirectly, linking *AtTOR* delayed flowering phenotypes and sensitivity to day length with flowering time control.

Following the establishment of the IFM, flower primordia are initiated at its flanks, producing a Floral Meristem (FM) and flanking floral organ primordia. Mutation of *lst8-1* produces flowers with smaller floral organs, but no changes in organ patterning or number have been reported (Moreau et al., 2012), suggesting that *LST8* may be independent from the ABC patterning genes (See review: Irish, 2017). Abnormal flower phenotypes have also been recorded in *raptor1* SALK lines, but not described fully (Anderson et al., 2005). Furthermore, *tor* knockdown flowers have yet to be investigated, and are necessary to determine whether flower development is under the control of *AtTOR* as well as *LST8* and *RAPTOR1*. It is unclear whether the phenotypes are due to direct changes to cell cycle/growth genes, and/or interactions with genes specific to floral development, such as *AUXIN RESPONSE FACTOR 8* (*ARF8*) and *BIGPETALp* (*BPEp*) (Szecsi et al., 2006; Varaud et al., 2011).

Interestingly, ectopic expression of Lily *S6K* (*LIS6K*) in *A. thaliana* produces flowers with shortened petals and stamens, due to reduced cell expansion and normal cell division (Tzeng et al., 2009). S6K is a conserved target of TOR; it was shown that S6K binds to RAPTOR for phosphorylation by TOR in plants (Mahfouz et al., 2006). S6K1 in turn phosphorylates the subunit h of eukaryotic Initiation Factor 3 (eIF3h), which promotes loading of mRNAs that carry upstream open reading frames (uORFs) within their 5' untranslated regions (5'UTRs) into the ribosome for translation re-initiation (**Figure 1**) (Schepetilnikov et al., 2013). Plant specific genes such as *AUXIN RESPONSE FACTORS* (*ARFs*) and *BASIC LEUCINE ZIPPER 11* (*bZIP11*) are encoded by uORF-mRNAs and therefore their translation reinititation may be under the control of TOR *via* S6K (Schepetilnikov et al., 2013). It would be interesting to investigate if ARFs with important roles in development such as *MONOPTEROS* (*ARF5*) and *ARF2* (Schruff et al., 2006; Chapman and Estelle, 2009) are activated by S6K in different tissue types such as the leaves and flower, and whether this S6K activation is dependent on TOR activity under different environmental conditions.

## Concluding Remarks and Future Perspectives

The TOR signaling pathway is vital to integrate information about the nutrient and energy status of cells and tissues to direct the appropriate developmental and physiological response (Dobrenel et al., 2016). Our understanding of plant TOR has boomed over the past years, with studies beginning to expand beyond the model plant Arabidopsis. Evidence is clearly emerging that TOR has a conserved regulatory role in photosynthetic organisms, acting in conjunction with the antagonist SnRK1 to adapt growth and metabolism according to nutrient and hormone signals. Developmental pathways are highly interconnected and it will be interesting to determine how these interact with TOR signaling in a tissue specific manner, particularly at later developmental stages and in novel plant species. The synthesis of such processes will require bioinformatic pathway analysis to build networks at the RNA expression, protein expression, and protein modification levels, for a complete understanding of TOR activity in each tissue. Crucially, these signaling pathways may be even more critical in plants than in animals and yeast, since plant immobility prevents their escape from hostile environments or nutrient scarcity, placing increased importance on their developmental plasticity in response to the environment.

## Author Contributions

KM drafted the manuscript and all authors revised it.

## Conflict of Interest

The authors declare that the research was conducted in the absence of any commercial or financial relationships that could be construed as a potential conflict of interest.
